# Screening for cryptococcal antigenemia using the lateral flow assay in antiretroviral therapy-naïve HIV-positive adults at an Ethiopian hospital clinic

**DOI:** 10.1186/s13104-015-1707-6

**Published:** 2015-11-23

**Authors:** Anton Reepalu, Taye T. Balcha, Tadele Yitbarek, Godana Jarso, Erik Sturegård, Per Björkman

**Affiliations:** Infectious Diseases Research Unit, Department of Clinical Sciences, Faculty of Medicine, Lund University, Ruth Lundskogs gata 3, 21428 Malmö, Sweden; Ministry of Health, P.O. Box 1234, Addis Ababa, Ethiopia; Adama Regional Hospital, Adama, Oromia Ethiopia; Clinical Microbiology, Regional and University Laboratories, Region Skåne, Jan Waldenströms gata 59, 20502 Malmö, Sweden

**Keywords:** Cryptococcal infection, HIV, ART-naïve, Ethiopia, Lateral flow assay

## Abstract

**Background:**

Since treatment for latent cryptococcal infection (CI) before starting antiretroviral therapy (ART) reduces mortality in HIV-infected subjects, screening for cryptococcal antigen (CrAg) in blood is recommended for individuals with CD4 cell counts < 100 cells/µL in regions with high CI prevalence. We assessed CrAg screening using the lateral flow assay in HIV-infected adults eligible for ART in central Ethiopia.

**Results:**

HIV-positive patients (age ≥ 18 years, CD4 cell count < 350 cells/μL and/or WHO stage IV, no current or previous ART) were recruited at Adama Regional Hospital, Ethiopia (February 2013 until March 2014). CrAg was determined in plasma by lateral flow assay. Among 129 included participants (median age 35 years, 64 % female) the median CD4 cell count was 210 cells/μL (interquartile range 110–309); 29 (23 %) had CD4 cell count < 100 cells/μL. Two (1.6 %) participants were CrAg-positive (CD4 cell counts 171 vs. 250 cells/µL), one of whom had clinically manifest cryptococcal meningitis at the time of testing.

**Conclusions:**

In contrast to two recent reports from Ethiopia, we found few cases of CI among ART-naïve adults. Our study, which is the first using lateral flow assay for CrAg screening in this country, illustrates the need of larger surveys of CI prevalence among ART-naïve patients before defining recommendations on CI screening.

## Findings

Cryptococcal infection (CI) is a major cause of disease and death among HIV-infected individuals, with the highest case burden in sub-Saharan Africa [1]. Despite increasing access to antiretroviral therapy (ART), mortality by cryptococcal meningitis (CM) remains high [1], partly explained by unmasking of latent CI in individuals with advanced immunosuppression at ART initiation [2]. The presence of cryptococcal antigen (CrAg) in blood is strongly associated with subsequent mortality after starting ART [3]. Administration of pre-emptive fluconazole therapy to CrAg-positive patients before starting ART has been shown to reduce the incidence of CM [4]. For this reason, the World Health Organization (WHO) recommends CrAg screening in ART-naïve persons with CD4 cell counts <100 cells/µl in regions with high prevalence of cryptococcal antigenemia [5].

CM is known to occur in Ethiopia, but the magnitude of this disease is unclear. Recently, two cross-sectional studies reported high prevalence of cryptococcal antigenemia, both in ART-naive subjects and in patients receiving ART [6, 7].

Here, we present findings of an assessment of CI screening using the lateral flow assay in ART-naïve patients at a large public ART clinic in Central Ethiopia.

##  Methods

Participants were recruited at the ART outpatient clinic at Adama Regional Hospital, Adama. HIV-positive subjects fulfilling criteria for ART initiation at the time of the study (CD4 cell count < 350 cells/μL and/or WHO stage IV) were eligible for inclusion. Additional inclusion criteria were: age ≥ 18 years, no current or previous ART, residence in the catchment area and written informed consent. Inclusion lasted from February 26 2013 until March 8 2014. The study received ethical approval from the ethical review boards at Lund University, Sweden, and the Federal Institute for Science and Technology, Addis Ababa, Ethiopia.

Study nurses collected demographic and clinical data (including physical examination) at inclusion, following a structured questionnaire. CD4 cell count, complete blood count and CrAg testing was performed at the Adama Regional Laboratory. Determination of CrAg was done on plasma and cerebrospinal fluid (if available), using the IMMY CrAg LFA (Immuno-Mycologics Ink, Norman, OK), following the manufacturer’s instructions. In case of clinically suspected CM and/or other serious conditions, a physician in the clinic (TY) was consulted. Lumbar puncture was performed for all patients with suspected meningitis. The outcome of participants was assessed through clinic registers at the end of the study.

## Results and discussion

Baseline characteristics of the 129 included subjects are shown in Table 1. Two (1.6 %) patients were CrAg-positive. The first case was a 38-year old woman (CD4 cell count 171 cells/μL) who presented with headache, cough, dyspnoea, constitutional symptoms and neck stiffness. Lumbar puncture showed meningitis with detectable CrAg. Despite fluconazole treatment she died 17 days after diagnosis. The second case was a 38-year old man (CD4 cell count 250 cells/μL); at the time of testing he did not show any clinical symptoms or signs compatible with cryptococcosis.

**Table 1:** Baseline characteristics of included participants

	Total (n = 129)
Age, median (IQR)	35 (28–39)
Female gender	83 (64)
Pregnant, n (% of females)	1 (1)
Rural residence	18 (11)
BMI—kg/m^2^, median (IQR)	20 (18–22)
MUAC—cm, median (IQR)	23 (21–26)
CD4 cell count—cells/μL, median (IQR)	210 (110–309)
CD4 cell count <100 cells/μL	29 (23)
CD4 cell count <200 cells/μL	57 (45)
Hemoglobin—g/dL, median (IQR)	12 (11–13)
Study nurse investigations	
Currently diagnosed with TB	6 (5)
WHO HIV disease stage	
1–2	58 (47)
3	60 (48)
4	6 (5)
Neck stiffness	4 (3.2)
Neurologic deficit	0 (0)
Confusion	0 (0)
Self-reported symptoms	
Fever	48 (37.5)
Night sweat	41 (32.3)
Weight loss	58 (45.0)
Fatigue	50 (40.3)
Cough	33 (25.6)
Diarrhea	19 (14.7)
Headache	64 (49.6)

*IQR* interquartile range, *BMI* body-mass index, *MUAC* mid-upper arm circumference

Data presented as n (%), if not stated otherwise

None of the participants were diagnosed with cryptococcosis during follow-up. At the end of the study, 96 (74.4 %) of participants remained in care. One (0.8 %) death was reported; 6 (4.7 %) patients had been transferred to other health facilities, whereas 26 (20.2 %) were lost to follow-up.

Management of common and severe opportunistic infections is a critical component of HIV care. CI screening in subjects starting ART may improve treatment outcome, but the indication for screening depends on the prevalence of CI in different settings. In our study population the prevalence of CI was low. In particular, only one case of asymptomatic CI was detected; the other CrAg-positive subject had clinically apparent meningitis at presentation.

Our findings differ from those presented in two recent reports from Ethiopia [6, 7], one of which was based in the same uptake area as our investigation [7]. In contrast to ours, both these studies included large proportions of patients receiving ART (271/369 [74 %] and 127/254 [50 %], respectively, [6, 7]). Furthermore, we performed CrAg testing using the lateral flow assay. Although both latex agglutination and lateral flow assays have high specificity, the lateral flow assay has higher sensitivity, and is preferred for field use since it is cheaper, requires less equipment and does not require refrigeration [5, 8]. Compared to the agglutination assay, which was used in the previous Ethiopian studies, the lateral flow assay can be performed as a point-of-care test. Evaluations of the lateral flow assay show excellent performance [9]. Therefore we consider false-negative reactions unlikely; this is further supported by the absence of incident cases of cryptococcal disease following ART initiation in our participants. Apart from differences with regard to ART status and degree of immunosuppression, geographical and health facility factors could explain the discordant rates of CI found in these three Ethiopian studies. Interestingly, Alemu et al. noted significantly higher prevalence of CI in one of two study sites in the Addis Ababa area [6], suggesting that the rate of CI can vary greatly even within the same region. The two cases of CI that occurred in our population were found in persons with CD4 cell counts > 100 cells/µL. This finding supports the results reported by Beyene et al. [7], illustrating that CI can occur also in persons with CD4 cell counts above the threshold level in current screening recommendations.

Our study does not corroborate previous reports of high rates of CI in Ethiopian HIV-infected adults. Although CI screening has been shown to be cost-effective in regions with CI prevalence above 3 % [3], each decision to implement any simple screening procedure in HIV programs in resource-limited settings may cause other initiatives not to be undertaken. Therefore, it is crucial that decisions on management guidelines are based on broad and well-documented basis. In our opinion, larger studies of treatment-naïve persons from different geographical regions of Ethiopia, and with comparison of diagnostic methods, are required before definite guidelines on testing and management of CI can be issued.
